# Liuweidihuang Pill Alleviates Inflammation of the Testis via AMPK/SIRT1/NF-*κ*B Pathway in Aging Rats

**DOI:** 10.1155/2020/2792738

**Published:** 2020-05-24

**Authors:** Yuxin Wang, Zhangjie Yang, Lingling Yang, Qian Zou, Shuai Zhao, Na Hu, Dongmei Chen, Ruiqin Cui, Huiming Ma

**Affiliations:** ^1^Key Laboratory of Fertility Preservation and Maintenance of Ministry of Education, Ningxia Medical University, Yinchuan 750004, China; ^2^Institute of Human Stem Cell Research, The General Hospital of Ningxia Medical University, Yinchuan 750004, China; ^3^School of Chinese Medicine, Ningxia Medical University, Yinchuan 750004, China; ^4^Department of Anatomy, Histology and Embryology, School of Basic Medical Science, Ningxia Medical University, Yinchuan 750004, China

## Abstract

Liuweidihuang Pill (LP) is a traditional Chinese herbal formula that is often used in clinical practice to treat kidney deficiency syndrome. The present study investigated the antiaging effects of LP in a D-galactose- (D-Gal-) induced subacute aging rat model. The study also attempted to explore whether anti-inflammatory mechanisms that underpin the antiaging effects are mediated by the AMPK/SIRT1/NF-*κ*B signaling pathway. Rats were subcutaneously injected with D-Gal at a dosage of 100 mg/kg/d for 8 weeks. Upon successful induction of aging in the rats, the animal was administered LP at 0.9 g/kg/d by gavage for 4 weeks. Proteins of the testis were subsequently examined by western blot analysis, and associated locations in the testicular tissue were determined by immunohistochemistry. We observed that LP exerted antiaging effects in aging rats following the activation of AMPK/SIRT1. It was also observed that LP inhibited the expression of NF-*κ*B, thereby further attenuating inflammation of the testis. Therefore, LP can alleviate inflammation of the testis via the AMPK/SIRT1/NF-*κ*B pathway in aging rats.

## 1. Introduction

The aging process involves a progressive decline in the function of tissues and organs and is closely related to the development and progression of a variety of diseases [1, 2]. However, many theories currently exist regarding the mechanisms that underpin aging. Indeed it has been suggested that DNA damage response [3], autophagy [4], telomerase length [5], and free radicals all play a part in the aging process [6]. Furthermore, studies have shown that senescence can result in proinflammatory phenotypes, including elevated levels of typical inflammatory markers such as interleukin-1 (IL-1) [7], IL-6, and tumor necrosis factor (TNF-*α*); increases in the levels of these markers can indicate senescence [8, 9]. The processes of aging and inflammation are closely related. The aging process is often accompanied by an imbalance of inflammatory homeostasis, which can lead to aging. Thus, some researchers have suggested that chronic inflammation can be used as a biomarker for aging [10]. It has previously been shown that the inflammatory response plays an important role in regulating aging [11]. The population of the world is rapidly aging, and therefore anti-aging has gradually become an issue of worldwide importance. This is especially important for male senescence, where a reduction in the pituitary-gonad axis response leads to a decline in testicular function resulting in impotence, premature ejaculation, erectile dysfunction, body fatigue, sweating, and other symptoms [12]. Studies have shown that in aging testicles, the levels of the aging-related indicators P19, P21, and P53 increase. Furthermore, the levels of superoxidase dismutase (SOD) activity and inflammatory cytokines TNF-*α*, IL-1, and IL-6 are also significantly increased in aging testicles [13].

In traditional Chinese medicine (TCM), it is believed that kidney deficiency (*Yin* or *Yang* deficiency) can accelerate the process of aging. However, LP, which was initially recorded during the time of the Song Dynasty (960–1279 AD), can nourish kidneys, thereby alleviating dizziness, tinnitus, and weakness. LP is a classical prescription in TCM and is composed of *Rehmannia* root (Shu Di, family: Scrophulariaceae) (32%), *Cornus officinalis Siebold* (Shan Zhu Yu, family: Cornaceae) (16%), Chinese yam (Shan Yao, family: Dioscoreaceae) (16%), *Alisma plantago-aquatica subsp* (Zhe Xie, family: Alismataceae) (12%), *Paeonia suffruticosa* (Mu Dan Pi, family: Paeoniaceae) (12%), and *Poria cocos* (Fu Ling, family: Polyporaceae) (12%), supplemented with honey. In TCM, LP is used to enhance male health and treat deficiencies pertaining to the kidney. However, the associated mechanisms of action are yet to be elucidated. In recent years, studies investigating LP have suggested that it has antiapoptotic [14] and antioxidative [15] effects while also alleviating insulin resistance [16]. Thus, in this study, we explored the effects of LP on the alleviation of inflammation in the testes in aging rats. Previous studies have demonstrated that one of the components of LP, *Rehmannia*, exhibits both antioxidative and anti-inflammatory effects in vitro [17, 18]. In addition, studies have also shown that yam polysaccharides and yam oligosaccharides extracted from Chinese yam both exhibit strong in vitro free radical-scavenging activity against hydroxyl radicals and superoxide radicals [19, 20].

Adenosine monophosphate-activated protein kinase (AMPK), the main sensor of cellular energy status [21], has been shown to activate silent information regulator of transcription 1 (SIRT1) [22]. It is widely believed that inflammation can be suppressed by the synergistic effects of AMPK and SIRT1 [23]. Furthermore, overexpression of SIRT1 can reduce the inflammatory response via nuclear factor kappa beta (NF-*κ*B) [24], and therefore AMPK/SIRT1/NF-*κ*B is regarded as a classical inflammatory signaling pathway.

The aforementioned studies revealed the potential of LP as an antiaging agent due to its ability to suppress inflammation. Thus, in this study, a D-galactose- (D-Gal-) induced subacute aging rat model was utilized to evaluate the therapeutic effects of LP in relation to inflammation. The study also attempted to investigate the role of the AMPK/SIRT1/NF-*κ*B signaling pathway in the anti-inflammatory effects of LP.

## 2. Materials and Methods

### 2.1. Ethical Approval

Sprague-Dawley (SD) male rats, purchased from the Laboratory Animal Center of Ningxia Medical University, were maintained in a specific pathogen-free (SPF) laboratory. The experiments performed herein were approved by the Animal Care Committee of the Ningxia Hui Autonomous Region (IACUC No. SCXK (Ning) 2015-0001 and NYLAC-2018-091). In order to abide by the 3R Principle of Animal Experiments, the number of rats was minimized so that the smallest number was experimentally used while still retaining statistical significance. Thus, we chose *n* = 10 for each group.

### 2.2. Materials

LP was procured from Beijing Tongrentang Technology Development Co. (Beijing, China). D-Gal, >99% pure, was purchased from Sigma-Aldrich (St. Louis, MO, USA). The antibody to p-AMPK was purchased from Cell Signaling Technology (Boston, USA), and the antibody to SIRT1 was ordered from Affinity Biosciences (USA). The antibody to NF-*κ*B (P65) was bought from BOSTER (Wuhan, China). The antibodies to AMPK, HO-1, IL-1, IL-6, and IL-10 were all purchased from Boiss (Beijing, China), and the antibodies to P21^Waf1/Cip1^, P16^INK4A^, and TNF-*α* were procured from Wanleibio (Shenyang, China). HRP-conjugated secondary antibodies were purchased from Boiss. The chemiluminescence reagents were from Affinity Biosciences (USA).

### 2.3. Experimental Design and Tissue Collection

Male SD rats (*n* = 30, 8 weeks old), with an initial weight of 230–255 g, were randomly divided into 3 groups, namely, the control group, the aging model group, and the treatment group. Both the aging model group and the treatment group were subcutaneously injected with D-Gal at 100 mg/kg/d for 8 weeks [25]; the control group was subcutaneously injected with the same volume of normal saline. During the last 4 weeks of subcutaneous injection of D-Gal, the treatment group underwent intragastric administration of 0.9 g/kg/d LP (LP was dissolved in water until a final concentration of 0.18 g/ml was achieved; the associated volume was determined using the “dose conversion coefficient table for animal and human body weight” [26]). The control group and the aging model group were given normal saline gavage during those 4 weeks. After 8 weeks, rats were sacrificed by euthanizing with a CO_2_ overdose, bilateral testicular tissue was taken, one side was fixed with 4% paraformaldehyde for histological analysis, and the other side was preserved at −80°C for western blot detection.

### 2.4. Western Blot

Excised testicular tissue, stored at −80°C, was homogenized by \ultrasonic equipment with protein lysis buffer and was subsequently incubated on ice for 10 min. The resultant supernatant was separated by centrifugation (14,000 rpm for 15 min at 4°C) and stored at −20°C. Processed samples were separated by 10% or 8% SDS-PAGE and transferred to PVDF membranes. The membranes were subsequently blocked by 5% no-fat milk for 1 h and were then treated overnight with primary antibodies at 4°C. Next, the membranes were incubated with HRP-conjugated secondary antibodies (1 : 15,000) for 1 h; they were subsequently developed using chemiluminescence reagents.

### 2.5. Histological Analysis

Testicular tissue was fixed with 4% paraformaldehyde for 12 h and was then dehydrated, cleared, embedded in paraffin, and sectioned into 5 *μ*m slices with a microtome (Leica, Germany). The slides were incubated with 3% hydrogen peroxide for 15 min and then blocked with normal goat serum for 1 h at 37°C. The slides were subsequently incubated for 12 h at 4°C with primary antibodies. Next, the slides were incubated with a secondary antibody, and color was developed with 3,3′-diaminobenzidine (DAB); and the slides were counterstained with hematoxylin. Hematoxylin and eosin (HE) staining was also performed. All images were captured by a microscope (Leica, Germany).

### 2.6. Statistical Analysis

SPSS v22.0 software was used for statistical processing of the relevant data. The results of the western blot were quantified and expressed as the mean ± SD. Data that met the criteria of normal distribution and homogeneity of variance were compared by one-way analysis of variance (ANOVA), whereas data that did not meet these criteria were compared by the rank test. Dunnett's *t* test was used to compare with the aging model group, and *P* < 0.05 was deemed to be statistically significant.

## 3. Results

### 3.1. Evaluation of Subacute Aging Model Rats

**Figure 1:**
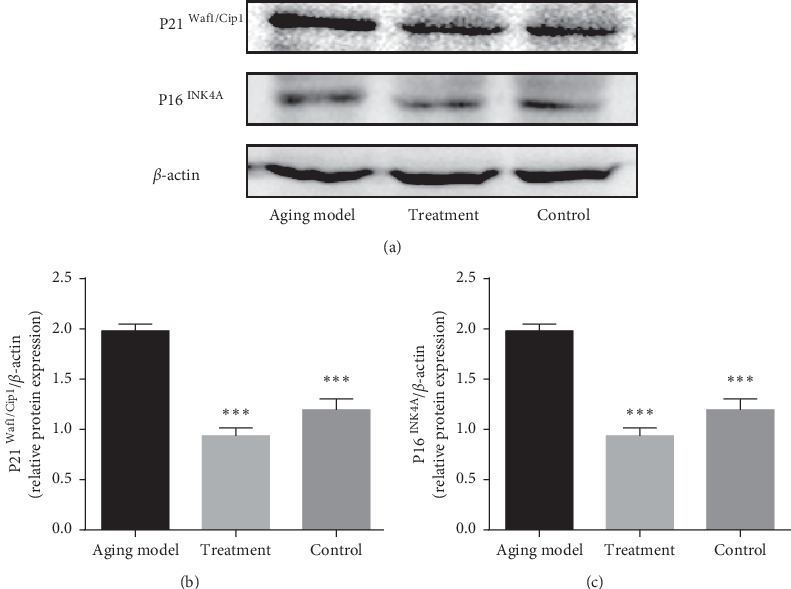
Evaluation of the efficacy of the aging model (subcutaneous injection of 100 mg/kg/d D-Gal for 8 weeks). The expression of aging-related proteins P16^INK4A^ and P21^Waf1/Cip1^ in the testicular tissue was detected by western blot. (a) Representative image; (b) quantification of P21^Waf1/Cip1^ expression; (c) quantification of P16^INK4A^ expression. *β*-actin was used as an internal loading control. Densitometry was used to compare expression levels. All data were expressed as the mean ± SD, *n* = 8 ^*∗∗∗*^*P* < 0.001, compared with the aging model.

The current study is based on a rat model of subacute aging where aging was induced by subcutaneous injection of D-Gal for 8 weeks; this strategy has been widely used to artificially age rodents for the purposes of research. The detection of aging-related proteins including P16^INK4A^ (multiple tumor suppressor 1, MTS) and P21^Waf1/Cip1^ (cyclin-dependent kinases inhibitor, CKI) indicated that the subacute aging model was successfully established (Figure 1(a)). The expression of P16^INK4A^ (*P* < 0.001) and P21^Waf1/Cip1^ (*P* < 0.001) in the testis were both significantly increased in the aging model compared with those of the control group. In addition, reduced expression of P16 ^INK4A^ (*P* < 0.001) and P21^Waf1/Cip1^ (*P* < 0.001) was observed in the testes of the rats that underwent intragastric LP administration compared with the aging model (Figures 1(b) and 1(c)).

**Figure 2:**
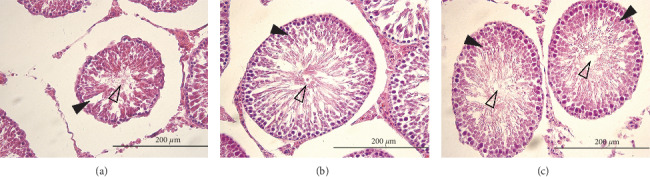
Histomorphological changes in the testicular tissue of the aging model (a), aging rats treated by LP (b), and control (c). Hematoxylin and eosin- (H&E-) stained testicular tissues of the rats are shown at 400x magnification. Black arrows indicate spermatogenic cells at various developmental stages, and hollow arrows indicate convoluted tubules where mature spermatozoa gather.

Histomorphology analysis of testicular tissue by HE staining indicated that the general structure of the testicular tissue in the aging model was more withered than that of the control and the treatment group, and the center of the convoluted seminiferous ducts exhibited distortion. A large number of spermatogenic epithelial germ cells were shed in the aging model group compared with the control group, with considerably reduced numbers of supporting cells and germ cells at all levels. The normal and treated rats had more matured spermatozoa in the lumen of the testicular tissue than the aging model (Figure 2).

### 3.2. LP Increases Antiaging Effects in Aging Rats via AMPK/SIRT1/NF-*κ*B Signaling Pathway

**Figure 3:**
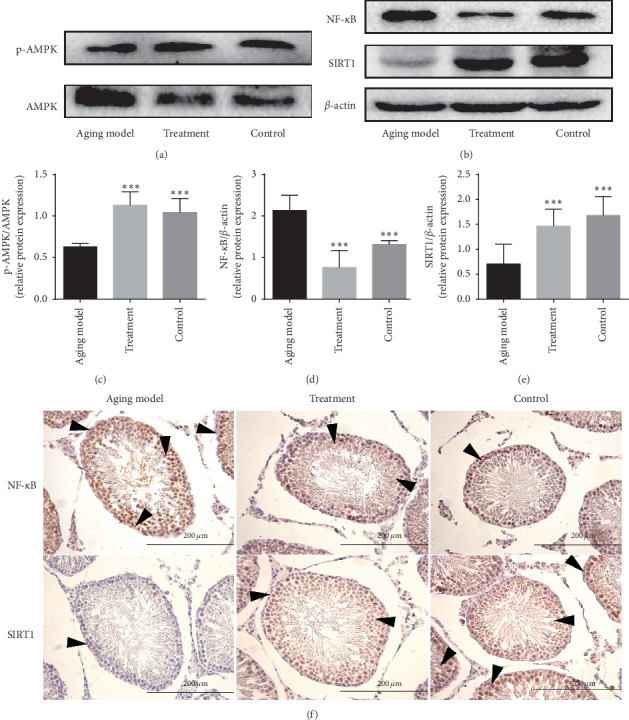
The effects of LP on anti-inflammation in testicular tissue of aging rats via AMPK/SIRT1/NF-*κ*B. (a) Representative western blot image showing the expression of p-AMPK and AMPK proteins; (b) representative western blot image showing the expression of AMPK, SIRT1, and NF-*κ*B protein; (c) quantification of the ratio of p-AMPK/AMPK; (d) quantification of NF-*κ*B expression; (e) quantification of SIRT1 expression. *β*-actin was used as an internal loading control. All data are expressed as the mean ± SD, *n* = 8. ^*∗*^*P* < 0.05, compared with the aging model. (f) Expression of NF-*κ*B and SIRT1 located in the testicular tissue was observed by immumohistochemical staining. Arrows indicate spermatogenic cells stained positive (magnification 400x).

Compared with the aging model, the expression of p-AMPK/AMPK (Figure 3(c)) and SIRT1 (Figure 3(e)) in the testes of the rats treated by LP was significantly elevated (*P* < 0.001). However, we observed that the expression of NF-*κ*B protein (Figure 3(d)) was significantly reduced (*P* < 0.001) in the treatment group compared with that in the aging model. Immunohistochemical staining showed an increase in the localization of NF-*κ*B in the testes of the aging model compared with that of the treatment group and the control group, while the location of SIRT1 in the testicular tissue showed an opposite trend (Figure 3(f)).

### 3.3. Effects of LP on Anti-Inflammation of Testes in Aging Rats

**Figure 4:**
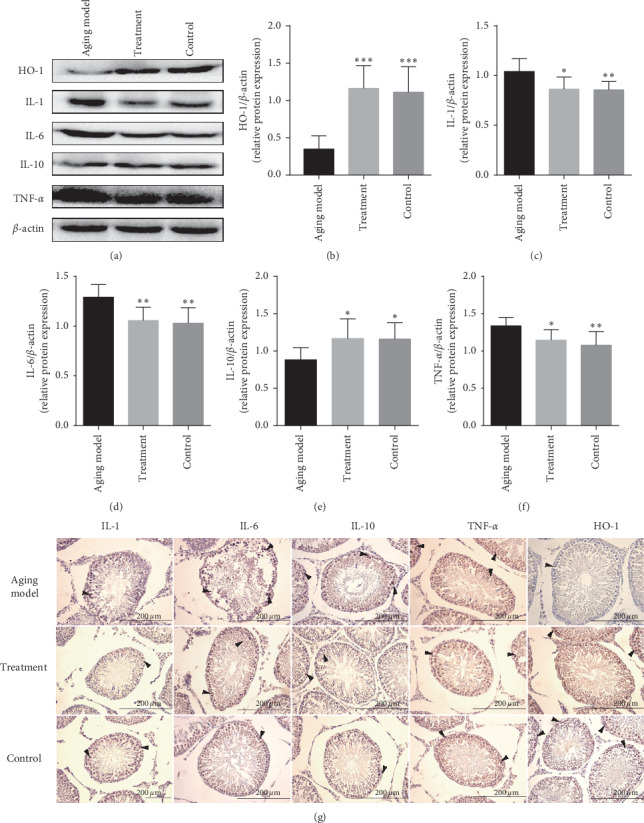
The effect of LP on anti-inflammation in the testicular tissue was demonstrated by western blot and immunohistochemical staining of testicular tissue of the control group, the aging model, and the treated group. Specific antibodies were used for the detection of IL-1, IL-6, IL-10, TNF-*α*, and HO-1. (a) Representative western blot image; (d–f) mean densities of HO-1, IL-1, IL-6, IL-10, and TNF-*α*. All data are expressed as the mean ± SD, *n* = 8. ^*∗∗∗*^*P* < 0.05, ^*∗∗*^*P* < 0.01, ^*∗∗∗*^*P* < 0.001 compared with the aging model. (g) Representative photographs of immunohistochemical staining. Arrows indicate spermatogenic cells stained positive (magnification 400x).

LP administration resulted in activation of the AMPK/SIRT1/NF-*κ*B signaling pathway in the testes of rats. Further investigation revealed that rats in the treatment group exhibited a mild reduction in the expression of proinflammatory cytokines IL-1 (*P* < 0.05), IL-6 (*P* < 0.01), and TNF-*α* (*P* < 0.05) compared with those from the aging model group. However, expression of IL-10 (*P* < 0.05) and HO-1 (*P* < 0.001) was significantly elevated in the treatment group compared with that of the aging model. Microscopic analysis by immumohistochemical staining revealed a pattern for IL-1, IL-6, TNF-*α*, IL-10, and HO-1 expression in the testicular tissue similar to that found by western blot analysis and showed that spermatogonia, sertoli cells, and spermatids were stained strongly (Figure 4).

## 4. Discussion

Extensive research has been performed in TCM on the topic of aging, and there has been a lot of discussion regarding changes pertaining to human body viscera function caused by the aging process. One of the more prominent theories in this field relates to the role of kidney function in the process of aging [27]. TCM posits that the kidney is the innate root of *Yin* and *Yang*. Thus, it is believed that many diseases are underpinned by deficiencies of the kidney. In general, it is believed in TCM that weakness of the kidney underpins the aging process [28]. LP is an ancient TCM prescription, which elicits kidney nourishing and tonifying effects [29]. In clinical treatment, kidney deficiency has been shown to result in lower back and knee pain, dizziness, and tinnitus, among other symptoms. In recent years, the scientific community has hypothesized that inflammation [30] and oxygen free radicals [31] are important effectors of senescence. Indeed, many studies have investigated anti-inflammation agents in relation to the process of aging [32]. In terms of inflammation, we compared the expression of inflammatory factors in the testes of an aging rat model along with rats treated with LP to analyze the effect of LP on antiaging.

In order to establish a suitable aging model in this study, the concentration of D-Gal utilized was carefully selected. A previous study reported that a concentration of D-Gal ranging from 50 mg/kg to 200 mg/kg should be maintained for 4–10 weeks [33], while 100–300 mg/kg of D-Gal is required for subcutaneous injection over a period of 4–8 weeks; this was deemed an appropriate model for the current experiment [34]. In this current study, an 8-week subcutaneous injection regimen of 100 mg/kg (a medium dosage) was utilized [25]. The subacutely aging rats were evaluated using aging-related biological indicators and histomorphology. A previous study reported P16^INK4A^ and P21^Waf1/Cip1^ as aging-related proteins [35]. Here, we observed that the expression of these two proteins in the testes of aging model rats was higher compared with that of the control group. This observation indicates that the process of aging is accelerated in the aging model rats. In addition, HE staining showed that the testicular tissue of aged rats was distorted, and the general structure of the testicular tissue in the aging model was more withered than that of the control group. Thus, the aging model was successfully established in this study.

It is well known that the AMPK/SIRT1/NF-*κ*B signaling pathway serves as an inflammatory pathway [36]. AMPK is considered an intracellular energy receptor that can rapidly sense energy deficiencies [37] in adverse environments, thereby facilitating the generation of an appropriate metabolic response to sustain cells [38]. The activation of AMPK, usually determined by the ratio of p-AMPK/AMPK [39], is involved in the regulation of a series of senescence-related signaling pathways such as SIRT1 [40], CRTC-1 [41], and mTOR [42]. AMPK also plays an important role in the aging process. SIRT1, a member of the sirtuin family, is similar to other family members and is homologous to SIRT2, a gene that is involved in the regulation of silencing information in yeast [43]. Studies have shown that SIRT1 can regulate pathophysiological processes such as inflammation, metabolism, cell proliferation, apoptosis, and senescence via deacetylation of substrates; SIRT1 also participates in multiple signaling pathways [44, 45]. NF-*κ*B, one of the members of the redox-sensitive transcription factors [46] of which P65 is an important member [47], can regulate the survival, differentiation, and proliferation of cells through the regulation of gene expression during tissue damage, stress, and inflammation [48]. A separate study suggested that the AMPK-SIRT1 pathway exhibits significant regulatory effects on inflammation [49]. Moreover, AMPK and SIRT1 can regulate transcription factor NF-*κ*B, which itself is involved in regulating the suppression of proinflammatory cytokines [50, 51]. In the treatment group, the ratio of p-AMPK/AMPK and the levels of protein SIRT1 in testicular tissue were both significantly elevated compared with those of the aging model. Furthermore, LP was shown to exhibit antiaging effects in the testes of the D-Gal-induced subacute aging model by activating AMPK/SIRT1; this event led to a significant reduction in the expression of NF-*κ*B.

Inflammatory factors, pathogens, bacteria, and oxidative stress can all initiate the NF-*κ*B inflammatory pathway [52]. Under normal physiological conditions, NF-*κ*B exists in the cytoplasm with I*κ*B*α* (nuclear factor of kappa light polypeptide gene enhancer in B-cell inhibitor) in an inactive state. When the body is stimulated by external stimuli, IKK (I*κ*B kinase) is activated, resulting in the IKK*β*-mediated phosphorylation of serine 32 and 36 of I*κ*B*α*. Phosphorylated I*κ*B*α* is subsequently ubiquitinated and degraded by the 26S proteasome, leading to the exposure of the nuclear localization region of the NF-*κ*B complex [53]. NF-*κ*B is then transferred from the cytoplasm to the nucleus, where it induces the expression of downstream proinflammatory factors genes such as IL-1, 1L-6, and TNF-*α* [54, 55]. The expression of proinflammatory factors IL-1, IL-6, and TNF-*α* was slightly reduced in the testes of rats treated with LP; however, the expression of IL-10 and HO-1 was higher in the LP-treated group compared with that in the aging rats. Thus, upon analysis of these results, it is apparent that the inflammatory response was slightly reduced in the treatment group because of the inhibition of NF-*κ*B by AMPK/SIRT1.

However, many effective Chinese medicinal compounds that are composed of mixtures of different herbs are yet to be fully characterized. With this in mind, we intend in the near future to analyze the active constituents of LP by the gas chromatography-mass spectrometer. The analysis of constituents of LP may identify compounds known to have antiaging effects, such as melatonin [56], tocopherol [57], polysaccharides [58], retinol [59], and tannins [60]. We ultimately hope to detect the effective constituents of LP in the serum of rats treated with optimal LP concentrations. It is hoped that this analysis will further reveal the effects of constituents of LP on antiaging.

## 5. Conclusion

In conclusion, LP exerts protective effects in relation to the expression of inflammatory cytokines in an aging rat model. The latter phenomenon involves the AMPK/SIRT1/NF-*κ*B signaling pathway. Our results suggest that LP exhibits potential as a therapeutic drug that targets inflammation during the process of aging. It is hoped that this analysis will provide a platform for future studies investigating the effects of LP on both inflammation and aging.

## Data Availability

The data used to support the findings of this study are included within the article.

## Abbreviation

LP: Liuweidihuang Pill
D-Gal: D-galactose
P-AMPK: Phosphorylation-adenosine monophosphate-activated protein kinase
SIRT1: Silent information regulator of transcription 1
NF-*κ*B: Nuclear factor kappa beta
SD: Sprague-Dawley
SPF: Specific pathogen-free
P16: Multiple tumor suppressor 1, MTS
P21: Cyclin-dependent kinase inhibitor, CKI
IL-1: Interleukin-1
IL-6: Interleukin-6
IL-10: Interleukin-10
TNF-*α*: Tumor necrosis factor -*α*
SOD: Superoxide dismutase
HO-1: Heme oxygenase 1
CRTC-1: CREB-regulated transcription coactivators
mTOR: Mammalian target of rapamycin
SIRT2: Silent information regulator of transcription 2
SDS-PAGE: Sodium dodecyl sulfate polyacrylamide gel electrophoresis
PVDF: Polyvinylidene fluoride
HE: Hematoxylin and eosin
DAB: Diaminobenzidine
TCM: Traditional Chinese medicine
I*κ*B*α*: Nuclear factor of kappa light polypeptide gene enhancer in B-cell inhibitor
IKK: I*κ*B kinase.
